# Nurse-led non-ICU early rehabilitation after liver transplantation: a quasi-experimental historical-control study

**DOI:** 10.3389/fpubh.2026.1812156

**Published:** 2026-10-23

**Authors:** XiaoFeng Xu, Jing Xu, Ting Zeng, Gang Xu, JiaYin Yang

**Affiliations:** 1 Liver Transplant Center, Organ Transplant Center, Department of General Surgery, West China Hospital of Sichuan University, Chengdu, China; 2 West China School of Nursing, Sichuan University, Chengdu, Sichuan, China; 3 Laboratory of Liver Transplantation, Institute of Organ Transplantation, Key Laboratory of Transplant Engineering and Immunology, NHC, Sichuan University, Chengdu, Sichuan, China

**Keywords:** early rehabilitation, liver transplant, non-ICU transition care, post-transplant service, quasi-experimental study

## Abstract

**Background:**

Early rehabilitation after liver transplantation is increasingly encouraged, but most programs are implemented after intensive care unit (ICU) stabilization. We evaluated a nurse-led pathway in which selected liver transplant recipients were transferred directly to the general ward after surgery. Because the intervention was introduced sequentially over time, the study was analyzed as a non-randomized historical-control quasi-experiment.

**Methods:**

Recovery-timing, device-duration, length-of-stay, and hospitalization-charge analyses were conducted in the 102 recipients who survived to complete postoperative outcome ascertainment (57 intervention and 45 control) and are therefore survivor-conditional. Continuous outcomes were assessed for distributional assumptions and compared using two-sided Mann-Whitney *U* tests when non-normal. Sparse binary outcomes were analyzed using Fisher's exact test. Multivariable linear regression with HC3 heteroscedasticity-robust standard errors adjusted for age, sex, body mass index, preoperative diagnosis, surgery duration, and log-transformed intraoperative blood loss. Log1p-transformed models were used as sensitivity analyses for skewed outcomes.

**Results:**

Compared with historical controls, recipients treated during the intervention period had earlier initiation of oral intake (median 13.0 vs. 48.0 h, *P* < 0.001), respiratory training (13.0 vs. 48.0 h, *P* < 0.001), and first out-of-bed activity (39.0 vs. 72.0 h, *P* < 0.001), as well as shorter urinary and central venous catheter retention (both *P* < 0.001). In adjusted models, intervention-period status was associated with earlier initiation of oral intake (adjusted difference −51.76 h, 95% CI −70.71 to −32.82), earlier respiratory training (−56.58 h, 95% CI −83.66 to −29.50), earlier ambulation (−60.48 h, 95% CI −103.24 to −17.72), shorter urinary catheter retention (−85.93 h, 95% CI −114.11 to −57.75), shorter central venous catheter retention (−66.17 h, 95% CI −89.70 to −42.63), and shorter length of stay (−4.48 days, 95% CI −8.23 to −0.73). Multidrug-resistant bacterial infection was numerically lower (3.5% vs. 13.3%) but not statistically significant (RR 0.26, 95% CI 0.06–1.24; Fisher's exact *P* = 0.134). Total hospitalization charges were lower in the unadjusted comparison but showed no statistically significant adjusted association with the pathway (*P* = 0.467).

**Conclusion:**

In this historical-control quasi-experimental study, implementation of the nurse-led non-ICU transition pathway was associated with earlier achievement of several postoperative rehabilitation milestones and shorter hospital stay after adjustment for measured covariates. Because intervention status was inseparable from calendar period and unmeasured temporal confounding could not be excluded, these findings should be interpreted strictly as associations rather than evidence of direct intervention effects. Prospective multicenter studies with contemporaneous controls and predefined safety criteria are needed before broader implementation.

## Introduction

Liver transplantation is currently the most effective treatment for end-stage liver disease (1). American scholar Thomas Starzl (2) completed the world's first liver transplantation in 1963, and China's liver transplantation started in the 1970s. With the development of medicine and the continuous improvement of liver transplantation treatment technology, the quantity and quality of liver transplantation in China have reached the international leading level (3). Data from the China Liver Transplantation Registry show that the average number of liver transplants in China was about 6,000 per year from 2018 to 2020 (4). Liver transplantation is complex, with trauma and a high incidence of postoperative complications (the incidence of postoperative complications is about 14%~35%), and nursing is complex and difficult (5, 6). Early intervention in patients with liver transplantation, in the enhanced recovery after surgery (ERAS) concept, under the guidance of the liver transplantation patients' early bed activity, can not only promote patients to restore gastrointestinal function as soon as possible, reduce the incidence of postoperative complications such as lower limb venous thrombosis, lung infection, also can improve the patient body coordination and self-care ability, improve sleep quality and postoperative fatigue (7, 8).

After liver transplantation, patients often have delayed functional recovery due to long-term bedridden, surgical trauma, and immunosuppression treatment (9). The traditional care model focuses on life support within the intensive care unit (ICU) but pays insufficient attention to the transition phase of early postoperative rehabilitation (10). The traditional concept is that liver transplant patients should have early life support in the ICU and strict bed rest 1 week after surgery for early activities. It could prevent bleeding in the liver section and anastomosis in the transplant area due to early implantation activities (11). However, sizeable surgical wounds, long bed braking time, and unstandardized and timely early activities will increase the incidence of complications, such as pulmonary infection, pleural effusion, pressure injury, lower limb venous thrombosis, which will lead to prolong the recovery time of liver transplant recipients and reduce their postoperative quality of life (12). Therefore, optimizing perioperative management strategies and promoting rapid and safe rehabilitation of patients is of great significance for improving patient survival rates after liver transplantation (13). Transplant center studies show improved postoperative monitoring and management can increase the 1-year survival rate from 72% to 79% to 85% to 90% and the 10-year survival rate from 53% to 66% (14).

Previous studies have reported associations between early rehabilitation and favorable recovery outcomes in liver transplant recipients (15). Patients are commonly encouraged to begin in-bed activity from the first postoperative day and, when clinically tolerated, to progress to out-of-bed activity on subsequent days (16). Earlier postoperative ambulation has also been associated with shorter ICU and hospital stays in critically ill and postoperative populations (17, 18). However, most existing liver-transplant rehabilitation studies have been conducted within an ICU-transition model. Longer ICU exposure has been associated with greater risk of multidrug-resistant bacterial infection and other healthcare-associated complications (19–21). Therefore, this study examined the associations between implementation of a nurse-led non-ICU transition early rehabilitation pathway and postoperative recovery outcomes in liver transplant recipients.

## Methods

### Design and setting

This single-center quasi-experimental study used sequential historical controls and combined a prospectively implemented 2024 pathway with retrospectively abstracted 2023 routine-care controls. The 2023 control cohort had already received standard ICU-transition care before the research intervention existed and was not exposed to any study intervention. Ethical approval was granted by the Biomedical Ethics Review Committee of West China Hospital, Sichuan University on April 2, 2024 (2024 Review No. 439), before implementation of the nurse-led non-ICU pathway in July 2024. The approved study framework included retrospective review of existing 2023 routine-care records. Written informed consent was obtained prospectively from intervention-period participants. No study intervention was applied retrospectively to the 2023 control cohort. The study was conducted in accordance with the Declaration of Helsinki, and reporting was aligned with STROBE recommendations.

### Participants

The participants come from a general tertiary-level hospital in Chengdu, southwestern China.

Researchers screen participants based on inclusion and exclusion criteria. The inclusion criteria include: (1) patients with the end-stage of liver disease and will undergo their first liver transplant surgery; (2) agree to participate in the study and sign the informed consent form. Exclusion criteria were as follows: (1) multiple liver transplantation operations and (2) patients with cognitive impairment, limb paralysis, fracture, and severe cardiopulmonary insufficiency.

The sample size calculation was calculated based on formula *N* = [(Zα+Zβ) ^2^^*^ (2 ^*^σ^2^)] /δ^2^, when α = 0.05, *Z*α = 1.96, β = 0.1, *Z*β = 1.28. After the literature review, when σ = 10.40 and δ = 7.99, *N* = 35.57. Considering 10% of cases, the final sample size was 40 subjects in each group. Finally, a total of 102 patients were recruited for this study.

Analysis populations: The full safety cohort comprised all 106 recipients who entered the two study periods (59 intervention-period and 47 historical-control recipients). Mortality was reported using these original denominators. The survivor analysis cohort comprised 102 recipients (57 and 45, respectively) who survived to complete postoperative outcome ascertainment. Recovery-milestone, device-duration, length-of-stay, and hospitalization-charge analyses therefore estimate survivor-conditional associations. Death was not coded as an ordinary missing value or assigned an artificial rehabilitation time.

### Group grouping process

The control period was July to December 2023, before implementation of the nurse-led non-ICU transition pathway. During this period, recipients routinely received postoperative life support in the ICU before transfer to the general ward. Patients meeting the eligibility criteria during this period constituted the historical control group.

The intervention period was July to December 2024. After implementation of the nurse-led non-ICU transition early rehabilitation pathway, eligible recipients were transferred directly to the general ward after surgery and received the nurse-led rehabilitation program. Because intervention status was completely determined by calendar period, a separate calendar-period (“year effect”) term could not be included in the same regression model without perfect collinearity. We therefore adjusted for measured baseline and perioperative covariates and explicitly considered residual secular trends as a limitation of the historical-control design.

### Intervention and control condition

Patients in the intervention group received an early rehabilitation program developed by the study team from literature and guideline recommendations and refined through expert consultation. Fourteen experts (four liver transplant physicians, six liver transplant nursing specialists, including three advanced practice nurses at organ transplant centers, two rehabilitation therapists, and two nutrition therapists) reviewed the draft protocol. Experts had more than 10 years of medical or nursing experience, more than 5 years of liver transplantation experience, a master's degree or above, and an intermediate or higher professional title. The final program included early feeding and nutritional support, respiratory training, progressive mobilization, rehabilitation exercises, and timely management of invasive catheters (Figure 1). Nurses used a structured implementation checklist. Patients remained eligible for unplanned ICU transfer whenever ICU-level support was clinically required. The study dataset recorded whether an unplanned ICU transfer occurred, but prospectively standardized physiologic red-flag thresholds for transfer were not captured as study variables; therefore, no retrospective thresholds were constructed for this revision.

**Figure 1:**
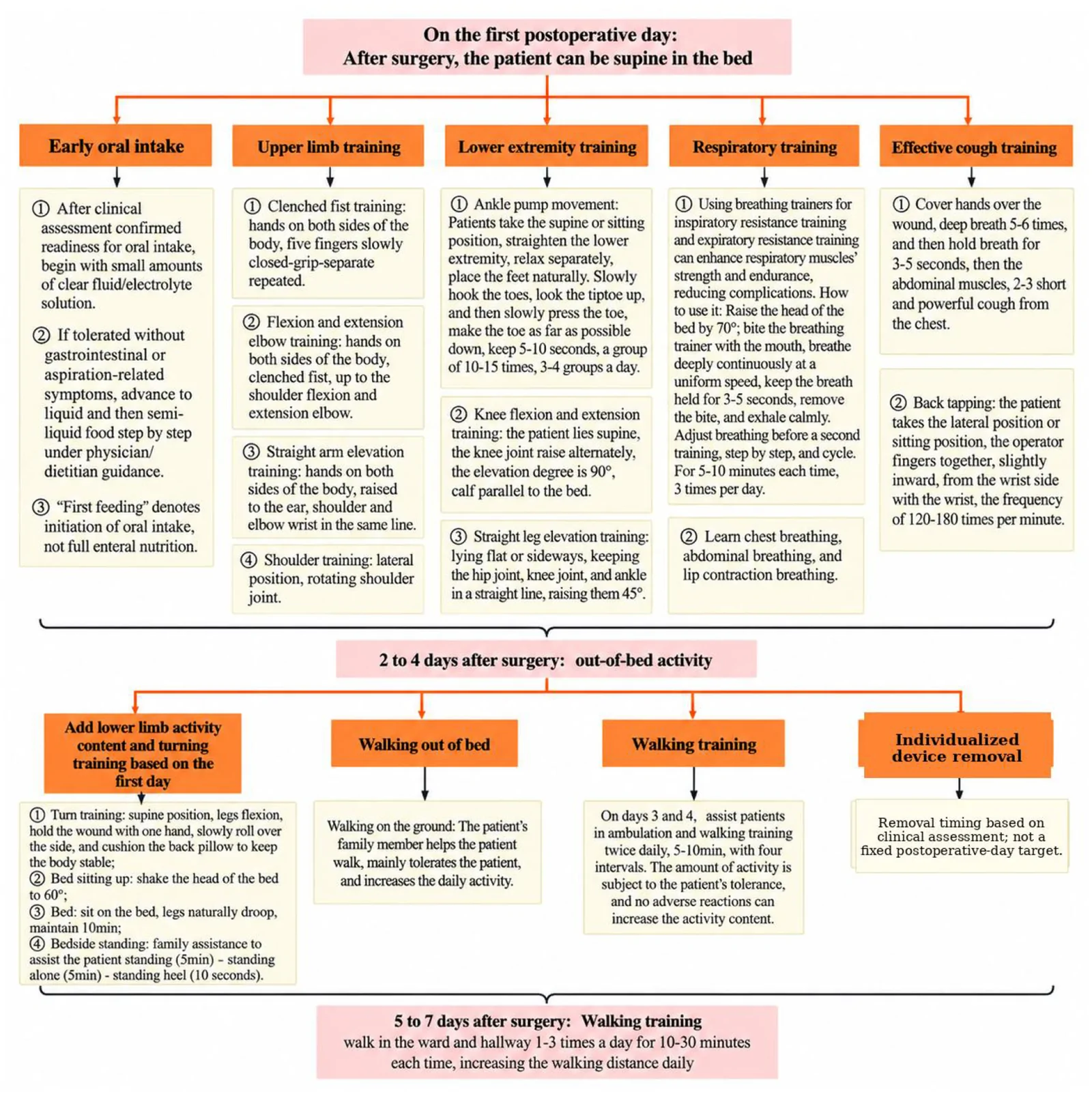
Nurse-led early rehabilitation program for non-ICU transition.

Ward monitoring, staffing, and escalation: recipients transferred directly from the operating area to the general ward were cared for in a dedicated level-II monitored unit established for immediate postoperative liver-transplant care. The nurse-to-patient ratio was approximately 1:3 during daytime shifts and 1:6 during night shifts. If a recipient's clinical condition changed or deteriorated, the medical team reassessed the patient and determined whether transfer to the ICU was required for continued treatment and organ-support needs. These arrangements describe the clinical procedures actually used during the intervention period; standardized physiologic escalation thresholds were not prospectively captured as study variables.

Nursing experience, airway management, and rehabilitation initiation/pausing: All nurses assigned to the level-II monitored unit had at least 6 months of prior ICU nursing experience. Tracheal extubation was performed only after physician assessment confirmed that the recipient met the clinical conditions for extubation. Initiation, progression, temporary suspension, and resumption of rehabilitation activities were determined according to joint clinical assessment by the treating physician and rehabilitation therapist. These assessments were individualized to the recipient's current clinical condition rather than governed by a retrospectively reconstructed set of fixed numeric thresholds.

Operational protocol for oral-intake progression: in this pathway, the term “early feeding” referred to initiation and staged advancement of oral intake rather than provision of complete enteral nutrition. After clinical assessment confirmed that oral intake could be attempted, patients began with small amounts of clear fluid/electrolyte solution. If this was tolerated without clinically important gastrointestinal intolerance or aspiration-related symptoms, oral intake was progressively advanced to liquid or semi-liquid food and subsequently toward a regular diet according to clinical tolerance and physician/dietitian assessment. Accordingly, the outcome “time to first postoperative feeding” represents time to initiation of oral intake and should not be interpreted as time to attainment of full caloric or protein requirements.

Participants in the historical control group received the routine postoperative pathway used before implementation of the non-ICU program. They were admitted to the ICU for early postoperative monitoring and life support, subsequently transferred to the general ward, and then received routine rehabilitation, nutrition, and catheter management according to usual physician and nursing orders. The structured non-ICU rehabilitation checklist was not used during the control period.

### Outcome variables and measures

Postoperative outcomes included time from surgery to initiation of oral intake, first respiratory function training, first out-of-bed activity, first flatus, and first defecation; recorded duration of the urinary catheter, central venous catheter, gastric tube, and arterial blood pressure (ABP) catheter; postoperative complication burden; multidrug-resistant bacterial infection; hospital length of stay (LOS); and total hospitalization charges in Chinese yuan (RMB). For gastric tubes and ABP catheters, review of the source records confirmed that a recorded duration of 0 h meant that the device was not inserted during the index hospitalization; it did not represent immediate removal or missing data. Accordingly, the primary duration variable for these two devices was interpreted as total device exposure per recipient, with non-insertion coded as 0 h. We additionally report device-placement proportions and retention duration conditional on actual insertion. LOS was defined as the total length of the index transplant hospitalization. The charge variable represented total nominal RMB billed during that admission; no inflation adjustment, separate pathway implementation costing, or cost-effectiveness analysis was performed.

Baseline variables available in the analytical dataset included preoperative diagnosis, age, sex, educational level, marital status, occupation, body mass index (BMI), surgery duration, and intraoperative blood loss.

### Data collection

For the 2023 historical-control period, demographic, operative, device, outcome, and resource-use variables were abstracted retrospectively from routine clinical records. For the 2024 intervention period, pathway implementation and study variables were documented contemporaneously and subsequently entered into the analytical dataset. A trained investigator performed data extraction/entry, and a second trained investigator reviewed the collected data for consistency. Mortality was retained as a safety outcome in the full cohort. All patient information was handled confidentially.

### Statistical analysis

Participant-level data were reanalyzed using Python 3.13, SciPy 1.17.0, pandas 2.2.3, and statsmodels 0.14.6. Continuous variables were summarized using medians and interquartile ranges (IQRs) when distributional assumptions were not met. Normality was assessed using the Shapiro-Wilk test and inspection of observed distributions. Because most recovery, device-retention, LOS, and charge variables were non-normal in at least one group, unadjusted between-period comparisons used two-sided Mann-Whitney U tests; rank-biserial correlation was reported as an effect-size measure. Categorical variables were compared using Pearson's chi-square test when appropriate, and Fisher's exact test was used for sparse binary outcomes with risk ratios (RRs) and 95% confidence intervals (CIs). Postoperative complication count was analyzed using the Mann-Whitney U test. Mortality was described in the full cohort of 106 recipients; recovery-timing, device-duration, LOS, and charge analyses were survivor-conditional among 102 recipients. For gastric-tube and ABP outcomes, 0 h denoted non-insertion. Therefore, the primary analyses for these devices estimated total exposure per recipient; secondary analyses reported insertion proportions and duration conditional on insertion.

Given the non-randomized historical-control design, adjusted analyses were performed using multivariable linear regression with HC3 heteroscedasticity-robust standard errors. The model specification for each continuous outcome was: outcome = intercept + intervention-period indicator + age + sex + BMI + diagnosis indicators + surgery duration + log1p(intraoperative blood loss). Intervention-period status was coded 1 for the intervention period and 0 for historical control; sex was coded as female vs. male; diagnosis was represented by two indicator variables with decompensated cirrhosis as the reference category and hepatic failure and hepatocellular carcinoma as comparison categories. The 102-patient survivor dataset had no missing values in model covariates or analyzed outcomes. Variance inflation factors were examined for multicollinearity, residual heteroscedasticity was assessed, and influence diagnostics (including Cook's distance) were reviewed. No observation was excluded solely on the basis of influence. Log1p-transformed outcome models were fitted as sensitivity analyses for all modeled continuous outcomes, with numerical results reported in Table 1. Because intervention status was identical to calendar period, the period effect could not be estimated separately from pathway status. Sparse infection and ICU-transfer outcomes were not multivariably adjusted to avoid unstable overfitted models.

**Table 1:** Log1p-transformed sensitivity analyses of adjusted continuous outcomes.

Outcome	*N*	Relative difference in (outcome + 1)^*^	95% CI	*P*
Initiation of postoperative oral intake	102	−71.3%	−78.1% to −62.4%	< 0.001
First respiratory training	102	−72.8%	−79.3% to −64.4%	< 0.001
First out-of-bed activity	102	−41.4%	−57.9% to −18.5%	0.0015
First flatus	102	+3.4%	−14.2% to +24.5%	0.726
First defecation	102	+5.6%	−14.5% to +30.3%	0.615
Urinary catheter retention	102	−77.3%	−82.9% to −69.7%	< 0.001
Central venous catheter retention	102	−68.0%	−76.6% to −56.3%	< 0.001
Gastric tube retention	102	−49.8%	−76.5% to +6.9%	0.0738
ABP catheter retention	102	−5.6%	−57.2% to +108.4%	0.887
Length of stay	102	−21.2%	−32.2% to−8.5%	0.0018
Total hospitalization charges	102	−1.3%	−13.2% to +12.2%	0.845

^*^Calculated as exp(beta)-1 from the log1p-transformed model and expressed as a percentage. Models used the same covariates as Table 7. These sensitivity analyses do not remove selection bias or unmeasured temporal confounding.

## Results

### Participant characteristics

A total of 106 liver transplant recipients entered the two study periods: 59 during the intervention period and 47 during the historical-control period (Figure 2). In-hospital mortality was 2/59 (3.4%) in the intervention period and 2/47 (4.3%) in the control period. In the intervention period, one recipient died on postoperative day 9 from sepsis and one died on postoperative day 16 from multiple organ dysfunction syndrome. In the historical-control period, one recipient died on postoperative day 32 from septic shock and one died on postoperative day 40 from multiple organ dysfunction syndrome. The remaining 102 recipients (57 intervention and 45 control) comprised the survivor analysis cohort for rehabilitation timing, device duration, LOS, and hospitalization charges. The patient-level file available for the present statistical reanalysis did not include sufficiently detailed pre-death milestone timing or resource-use trajectories for the four decedents; accordingly, no artificial milestone times were assigned and no competing-event model was constructed. Measured baseline and perioperative characteristics in the survivor cohort were generally comparable between periods. Median surgery duration was 298 min (IQR 264–371) in the intervention group and 320 min (IQR 299–342) in controls (*P* = 0.361). Because of the non-randomized historical-control design, adjusted analyses nevertheless included prespecified clinically relevant covariates, including surgery duration and intraoperative blood loss (Table 2).

**Table 2:** Characteristics of the participants.

Characteristic	Intervention (*n* = 57)	Control (*n* = 45)	*P*
Male sex, *n* (%)	48 (84.2)	30 (66.7)	0.066
Age, years, median (IQR)	53 (47–57)	52 (39–57)	0.395
Married, *n* (%)	53 (93.0)	41 (91.1)	0.941
Employed, *n* (%)	21 (36.8)	19 (42.2)	0.846
College/university or above, *n* (%)	21 (36.8)	16 (35.6)	0.739
Han ethnicity, *n* (%)	52 (91.2)	37 (82.2)	0.291
BMI, kg/m^2^, median (IQR)	23.53 (22.32–25.82)	23.14 (20.96–25.88)	0.290
Decompensated cirrhosis, *n* (%)	33 (57.9)	23 (51.1)	0.409^*^
Hepatic failure, *n* (%)	7 (12.3)	10 (22.2)	
Hepatocellular carcinoma, *n* (%)	17 (29.8)	12 (26.7)	
Surgery duration, min, median (IQR)	298 (264–371)	320 (299–342)	0.361
Intraoperative blood loss, mL, median (IQR)	800 (600–1,000)	1,000 (600–1,300)	0.554

^*^The *P* value represents the overall comparison of preoperative diagnosis distributions between the intervention and control groups using Pearson's chi-square test.

**Figure 2:**
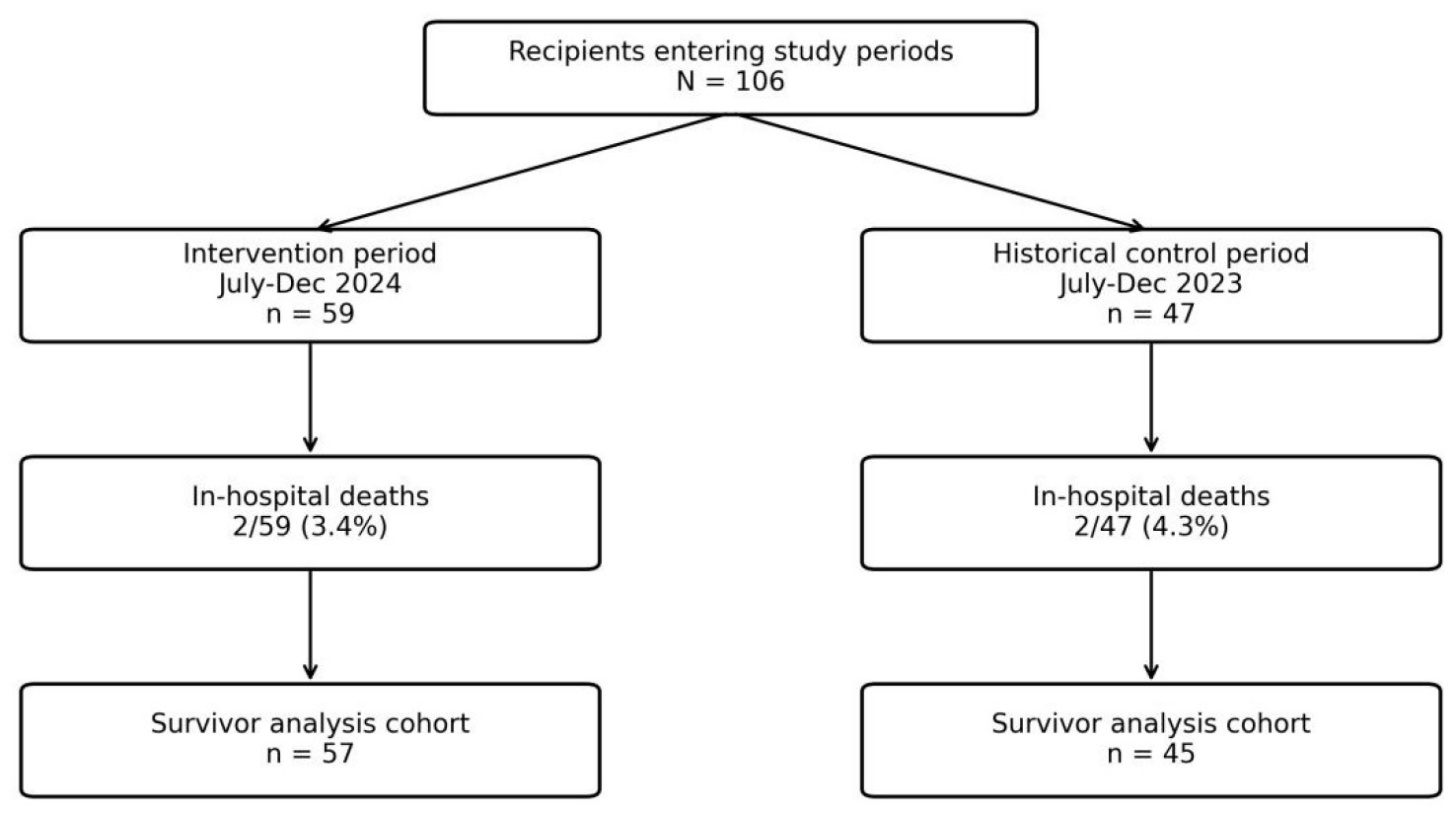
Participant flow and analysis populations. The full safety cohort included all 106 recipients entering the two study periods; survivor outcome analyses included 102 recipients who survived to complete postoperative outcome ascertainment. Intervention-period deaths occurred on postoperative days 9 (sepsis) and 16 (multiple organ dysfunction syndrome); historical-control deaths occurred on postoperative days 32 (septic shock) and 40 (multiple organ dysfunction syndrome).

### Associations between pathway implementation and early rehabilitation outcomes

Within the 102-recipient survivor analysis cohort, distributional assessment showed that most postoperative timing variables were non-normally distributed in at least one group; therefore, primary unadjusted comparisons used Mann-Whitney U tests. Recipients treated during the intervention period had earlier initiation of oral intake, respiratory training, first out-of-bed activity, urinary catheter removal, and central venous catheter removal than historical controls. These between-period differences are survivor-conditional descriptive associations and do not establish that the pathway itself caused the observed changes. Differences in time to first flatus, first defecation, gastric-tube duration, and ABP-catheter duration were not statistically significant under the distribution-appropriate non-parametric analysis (Table 3). In exploratory analyses restricted to recipients in whom these devices were actually inserted, median gastric-tube and ABP-catheter retention durations were shorter during the intervention period than among historical controls (Table 4). These conditional comparisons should be interpreted cautiously because device-insertion indications may have differed between periods

**Table 3:** Comparison of outcome indicators of early rehabilitation between the patients.

Outcome	Intervention, median (IQR)	Control, median (IQR)	*P*	Rank-biserial r
Initiation of postoperative oral intake, h	13.0 (10.0–15.0), *n* = 57	48.0 (27.0–72.0), *n* = 45	< 0.001	−0.853
First respiratory training, h	13.0 (11.0–15.5), *n* = 57	48.0 (45.0–72.0), *n* = 45	< 0.001	−0.873
First out-of-bed activity, h	39.0 (36.0–55.0), *n* = 57	72.0 (48.0–96.0), *n* = 45	< 0.001	−0.579
First flatus, h	57.5 (50.3–70.0)	48.0 (48.0–72.0)	0.134	0.173
First defecation, h	81.0 (68.0–105.5)	72.0 (48.0–96.0)	0.207	0.146
Urinary catheter retention, h	16.0 (14.0–24.0)	96.0 (54.5–120.0)	< 0.001	−0.887
Central venous catheter retention, h	22.5 (17.0–38.0)	72.0 (48.0–120.0)	< 0.001	−0.820
Gastric tube retention, h	0.0 (0.0–0.0)	0.0 (0.0–0.0)	0.156	−0.114
ABP catheter retention, h	22.5 (9.3–37.0)	34.5 (0.0–96.0)	0.250	−0.131

Device-placement and conditional device-duration analyses: Review of the source records confirmed that zero-hour values for gastric tubes and ABP catheters represented devices that were not inserted, rather than devices that were immediately removed or missing records. Accordingly, the primary analyses in Table 3 treat these values as zero device exposure per recipient. Gastric tubes were inserted in 9/57 (15.8%) intervention recipients and 11/45 (24.4%) historical controls; ABP catheters were inserted in 44/57 (77.2%) and 26/45 (57.8%), respectively. Among recipients in whom these devices were actually inserted, conditional retention durations are shown in Table 4. These conditional comparisons remain exploratory because the indications for device insertion may have differed between care periods.

**Table 4:** Exploratory conditional device-duration analyses.

Device	Inserted, intervention	Inserted, control	Median duration among inserted, intervention	Median duration among inserted, control	*P* ^*^
Gastric tube	9/57 (15.8%)	11/45 (24.4%)	13.0 (10.0–35.0) h	102.5 (42.0–192.0) h	0.0078
ABP catheter	44/57 (77.2%)	26/45 (57.8%)	34.0 (19.5–38.7) h	72.0 (48.0–120.0) h	< 0.001

^*^Two-sided Mann-Whitney U test among recipients in whom the corresponding device was inserted. For gastric tubes and ABP catheters, 0 h was confirmed to mean no device insertion; there were no immediate-removal or missing records coded as 0 h for these two variables. Conditional analyses should still be interpreted cautiously because device-insertion indications may have differed between care periods.

### Associations with postoperative complications and unplanned ICU transfer

Among the 102-recipient survivor analysis cohort, the median number of recorded postoperative complications was 1 (IQR 1–2) in the intervention group and 2 (IQR 1–3) in the control group (*P* = 0.283). Unplanned ICU transfer occurred in 2 of 57 intervention survivors (3.5%) and 2 of 45 control survivors (4.4%; Fisher's exact *P* = 1.000). Multidrug-resistant bacterial infection occurred in 2 of 57 intervention survivors (3.5%) and 6 of 45 controls (13.3%). Although the point estimate favored the intervention pathway, the confidence interval was wide and crossed the null (RR 0.26, 95% CI 0.06–1.24; Fisher's exact *P* = 0.134) (Table 5). In-hospital mortality for the full safety cohort was 2/59 (3.4%) vs. 2/47 (4.3%), as shown in Figure 2.

**Table 5:** Comparison of outcome indicators of postoperative complications between the patients.

Outcome	Intervention	Control	Test	*P*	Effect estimate (95% CI)
Postoperative complication count	1 (1, 2), *n* = 57	2 (1–3), *n* = 45	Mann-Whitney U	0.283	
Unplanned ICU transfer	2/57 (3.5%)	2/45 (4.4%)	Fisher exact	1.000	RR 0.79 (0.12–5.39)
Multidrug-resistant bacterial infection	2/57 (3.5%)	6/45 (13.3%)	Fisher exact	0.134	RR 0.26 (0.06–1.24)

### Associations with hospital length of stay and hospitalization charges

Length of stay and total hospitalization charges were right-skewed. These outcomes were analyzed in the 102-recipient survivor cohort. In unadjusted between-period comparisons, both measures were lower during the intervention period: median LOS was 13 days (IQR 11–16) vs. 17 days (IQR 14–23; *P* < 0.001), and median total hospitalization charges were RMB 400,381.50 (IQR 390,039.64–409,537.43) vs. RMB 432,475.80 (IQR 376,589.00–465,566.86; *P* = 0.016) (Table 6). These unadjusted differences should not be interpreted as direct effects of the pathway or as evidence of cost-effectiveness.

**Table 6:** Comparison of LOS and hospitalization charges between the patients.

Outcome	Intervention, median (IQR)	Control, median (IQR)	*P*	Rank-biserial r
Length of stay, days	13.0 (11.0–16.0), *n* = 57	17.0 (14.0–23.0), *n* = 45	< 0.001	−0.453
Total hospitalization charges, RMB	400,381.50 (390,039.64–409,537.43), *n* = 57	432,475.80 (376,589.00–465,566.86), *n* = 45	0.016	−0.279

### Adjusted analyses

Because the study used non-randomized sequential historical controls and several outcomes were heteroscedastic, multivariable linear models were fitted with HC3 heteroscedasticity-robust standard errors in the 102-recipient survivor cohort. Models adjusted for age, sex, BMI, preoperative diagnosis, surgery duration, and log-transformed intraoperative blood loss. Intervention-period status was associated with earlier initiation of oral intake, earlier respiratory training, earlier first out-of-bed activity, shorter urinary and central venous catheter retention, and shorter LOS. Adjusted associations for first flatus and first defecation were not statistically significant and are now reported in Table 7. Total hospitalization charges did not show a statistically significant adjusted association. Gastric-tube duration had a 95% CI crossing zero and *P* = 0.0504 and was not interpreted as statistically significant. For gastric-tube and ABP outcomes, 0 h represented non-insertion; therefore, the raw-scale coefficients estimate total device exposure per recipient and combine differences in both device placement and duration. For first out-of-bed activity, the group means were 43.8 +/− 18.7 h and 100.5 +/− 123.1 h, while the medians were 39 and 72 h; the adjusted coefficient (−60.48 h) estimates a covariate-adjusted mean difference and is therefore not expected to equal the median contrast. The largest Cook's distance was 0.94 in the ambulation model; no observations were excluded. Log1p-transformed sensitivity analyses are reported numerically in Table 1 and supported the direction and statistical significance of the adjusted associations for oral-intake initiation, respiratory training, ambulation, urinary catheter retention, central venous catheter retention, and LOS, but not for ABP catheter duration, gastric-tube duration, first flatus, first defecation, or total hospitalization charges. Maximum VIFs were below 1.3. Because calendar period and pathway status were identical, the adjusted coefficients cannot separate pathway-related associations from unmeasured secular changes.

**Table 7:** Adjusted associations between the non-ICU pathway and selected outcomes.

Outcome	*N*	Adjusted difference^*^	95% CI	*P*
Initiation of postoperative oral intake, h	102	−51.76	−70.71 to −32.82	< 0.001
First respiratory training, h	102	−56.58	−83.66 to −29.50	< 0.001
First out-of-bed activity, h	102	−60.48	−103.24 to −17.72	0.006
First flatus, h	102	−6.03	−27.56 to 15.51	0.583
First defecation, h	102	−1.79	−25.32 to 21.74	0.881
Urinary catheter retention, h	102	−85.93	−114.11 to −57.75	< 0.001
Central venous catheter retention, h	102	−66.17	−89.70 to −42.63	< 0.001
Gastric tube retention, h	102	−31.82	−63.68 to 0.05	0.0504
ABP catheter retention, h	102	−26.11	−43.00 to −9.21	0.002
Length of stay, days	102	−4.48	−8.23 to −0.73	0.019
Total hospitalization charges, RMB	102	−16,153	−59,685 to 27,379	0.467

^*^Adjusted mean difference for intervention period minus historical control from linear regression with HC3 robust standard errors. Models included age, sex, BMI, preoperative diagnosis (decompensated cirrhosis as reference), surgery duration, and log-transformed intraoperative blood loss. Negative values indicate earlier/lower outcomes during the intervention period. Analyses are survivor-conditional (*N* = 102). For gastric-tube and ABP outcomes, 0 h denotes no device insertion; coefficients therefore reflect total device exposure per recipient rather than retention time conditional on placement. Conditional duration analyses among inserted recipients are reported separately in Table 4.

## Discussion

In this quasi-experimental historical-control study, treatment during the nurse-led non-ICU pathway period was associated with earlier initiation of oral intake, respiratory training, and first out-of-bed activity, shorter urinary and central venous catheter retention, and shorter LOS after adjustment for measured baseline and perioperative factors among survivors. These associations are compatible with the intended sequence of the ERAS-oriented pathway, but the present design cannot determine whether the pathway itself caused the observed differences. Mortality was similar in the full safety cohort (2/59 vs. 2/47), but the principal recovery and resource analyses are survivor-conditional because detailed pre-death milestone and resource trajectories were not available in the patient-level reanalysis file. For gastric tubes and ABP catheters, zero-hour values were confirmed to represent non-insertion; thus, the corresponding primary duration analyses reflect total device exposure per recipient, while the conditional analyses among inserted recipients may still be influenced by between-period differences in device-placement indications. Because the pathway was implemented as a bundled care process and calendar period was completely confounded with pathway status, secular changes in perioperative practice, staffing, monitoring, discharge policy, or patient selection may partly or wholly explain the observed associations.

With the increasing maturity of liver transplantation in China, postoperative recovery has become an important component of transplant care (22, 23). Previous studies and guidelines support early mobilization after major surgery and transplantation (24–26). However, evidence remains limited for rehabilitation pathways implemented without routine ICU transition. In prior literature, prolonged ICU exposure has been associated with infection risk, immobilization, invasive-device exposure, and greater resource use (20, 27, 28). In the present study, earlier out-of-bed activity was observed during the intervention period and was more consistent with contemporary recommendations for early postoperative mobilization. This observation should nevertheless be interpreted as an association between care period and recovery timing rather than proof that bypassing routine ICU transition directly accelerated ambulation.

Multidrug-resistant bacterial infection was numerically less frequent in the intervention survivor cohort (3.5% vs. 13.3%), but the number of events was small and Fisher's exact test was not statistically significant. Likewise, postoperative complication count did not differ significantly. Unadjusted LOS and total hospitalization charges were both lower during the intervention period. After covariate adjustment, intervention-period status remained associated with shorter LOS in both the raw-scale HC3 model and the log1p sensitivity model, whereas total hospitalization charges showed no statistically significant adjusted association. The charge confidence interval was compatible with both lower and higher charges, so these data neither establish a cost reduction nor demonstrate equivalence. No formal cost-effectiveness analysis was performed, and additional ward staffing, training, and pathway implementation costs were not separately costed.

From a public-health perspective, the observed associations may inform hypotheses about postoperative care organization after liver transplantation, but they do not demonstrate improved functional recovery, cost savings, ICU-bed availability, or policy-scale efficiency. Earlier initiation of care activities is a process outcome and should not be equated with improved quality of life or long-term function. The exposure combined care location, feeding, rehabilitation, device management, and nursing coordination; the present study cannot isolate effects attributable specifically to nursing leadership or ICU bypass. Because calendar period and intervention status were inseparable, secular changes in infrastructure, operative technique, staffing, infection prevention, monitoring, nutrition practice, or discharge policy may have contributed to the between-period differences.

This study has several limitations. First, it was a single-center, non-randomized study using sequential historical controls; calendar period was completely confounded with intervention status, so residual secular confounding remains possible despite multivariable adjustment. Second, the main recovery, device-duration, LOS, and charge analyses were survivor-conditional; four in-hospital deaths were retained in the safety cohort, but the patient-level file used for this reanalysis did not contain sufficiently detailed pre-death milestone/resource trajectories for a competing-event analysis. Third, MELD scores and ASA physical-status classifications were not systematically available, preventing additional severity adjustment. Fourth, the decision threshold for direct ward management may have evolved over time, creating potential selection bias toward fitter recipients. Fifth, for gastric tubes and ABP catheters, zero-hour values represented non-insertion. Consequently, the primary duration analyses for these devices capture total device exposure per recipient rather than retention conditional on placement; the separate conditional analyses among inserted recipients may be affected by different insertion indications between periods.

In conclusion, among survivors, treatment during the nurse-led non-ICU transition early rehabilitation period was associated with earlier initiation of oral intake, respiratory training, and first ambulation, shorter urinary and central venous catheter retention, and shorter LOS after adjustment for measured covariates. Mortality was 2/59 vs. 2/47 in the full safety cohort. Total hospitalization charges and multidrug-resistant bacterial infection did not provide evidence of independent pathway-related benefit. Given the historical-control design, survivor-conditional analysis, between-period differences in device-placement practice for gastric-tube and ABP outcomes, and potential temporal and selection biases, all observed differences should be interpreted as associations rather than causal effects. Prospective multicenter studies with contemporaneous controls, explicit device-placement status, prespecified safety criteria, and formal economic evaluation are warranted before broad implementation.

## Data Availability

The raw data supporting the conclusions of this article will be made available by the authors, without undue reservation.
